# ﻿The diversity of Acarosporaceae (Acarosporales, Lecanoromycetes) in California

**DOI:** 10.3897/mycokeys.112.138580

**Published:** 2025-01-16

**Authors:** Kerry Knudsen, Jessica Cho-Ah-Ying, Jana Kocourková, Eva Hodková, Jiří Malíček, Yan Wang

**Affiliations:** 1 Department of Ecology, Faculty of Environmental Sciences, Czech University of Life Sciences, Prague, Kamýcká 129, Praha - Suchdol, 165 00, Czech Republic Czech University of Life Sciences Prague Czech Republic; 2 Department of Ecology and Evolutionary Biology, University of Toronto, Toronto, ON, Canada University of Toranto Torоnto Canada; 3 Institute of Botany, The Czech Academy of Sciences, Zámek 1, CZK 252 43 Průhonice, Czech Republic Czech Academy of Sciences Průhonice Czech Republic; 4 Department of Biological Sciences, University of Toronto Scarborough, Toronto, ON, Canada University of Toronto Scarborough Toronto Canada

**Keywords:** Channel Island National Park (Santa Rosa Island), Chihuahuan Desert, Joshua Tree National Park, Mojave Desert, San Bernardino Mountains, San Jacinto Mountains, Sonoran Desert

## Abstract

*Acarosporaalba*, *A.indistincta*, *A.sharnoffii*, and *A.tejonensis* are described from California. *Sarcogynefasciculata* is described from California and New Mexico. *Sarcogynecoeruleonigricans* is reported new for California. *Sarcogynelapponica* is recognized as a synonym of *Acarosporalapponica*, its basionym. We report 127 described species of Acarosporaceae for North America. We verified 62 species of Acarosporaceae from California.

## ﻿Introduction

The order Acarosporales contains a single family Acarosporaceae circumscribing seven genera of crustose lichens with green eukaryotic photobionts and polyspored asci (most with a hundred ascospores or more): *Acarospora*, *Lithoglypha*, *Myriospora*, *Pleopsidium*, *Sarcogyne* (including *Glypholecia*), *Timdalia* and *Trimmatothelopsis* (Knudsen et al. 2023a). We have failed to sequence *Lithoglypha* which is only known from the type collections and cannot verify if it is a monophyletic genus (Brusse 1988). The other six genera have various ascus stains. The most common is the *Acarospora*-type with IKI- tholus in *Acarospora*, *Lithoglypha*, *Myriospora*, *Sarcogyne* and most species of *Trimmatothelopsis*. An IKI+ blue tholus occurs in *Pleopsidium*, *Timdalia*, and in seven species of *Trimmatothelopsis*.

Acarosporaceae occur in a variety of habitats from seashores to mountain tops, but the family is most common in arid habitats (Magnusson 1930, 1933, 1940, 1944, 1952; Golubkova 1981; Nurtai et al. 2017; Leavitt et al. 2018; Jung et al. 2019; Knudsen et al. 2021a, 2023a). No species occur in the tidal zones, where lichens are submerged during high tide. No species are reported from the deep shade of dense forests. They do not occur in tropical biotopes such as the Amazon rainforest or the jungles of Africa but are common in arid South Africa or the Andes in South America (Magnusson 1933; Knudsen et al. 2008, 2012). Most species occur on calcareous or non-calcareous rock, with some species restricted to basic HCl- rock. Several species occur only in soil crusts, like *Acarosporanodulosa* and *A.schleicheri*. Several species have been reported on wood of which only *A.similis* in Europe appears to be an obligate (Stordeur et al. 2023). The most common species on wood in North America is *A.americana* (Knudsen 2021). Some saxicolous species are either facultative or obligate lichenicolous lichens, which begin as juvenile non-lichenized lichenicolous fungi, expropriating the algae of the host, and eventually morphing out of the host as distinctly different lichenized lichens, like the brown *A.interjecta* which is parasitic on the yellow *A.novomexicana* (Knudsen et al. 2023a, see fig. 10). A few species are lichenicolous fungi, not becoming lichenized, for instance *A.destructans* common in southern California and *A.lendermeri* on *Candelariella* species in Asia and western North America (Knudsen and Kocourková 2020; Knudsen et al. 2022a). Many species are pioneers, colonizing new substrate. Though Acarosporaceae can occur like many crustose lichens on anthropogenic substrates such as stone walls and gravestones, *Sarcogynepruinosa* (syn. *S.regularis*) is common on concrete and *A.moenium* on a variety of urban substrates. There are no sterile leprose taxa. There is one sorediate species, white with black soralia, *A.moenium*. Pycnidia do not occur in all species but have amazing variety in *Trimmatothelopsis* (Knudsen et al. 2023b). Replication by division of the thallus and apothecia is common. Several species like *A.applanata* from southwestern North America or *A.fissa* from the Czech Republic, are profusely crosshatched by abscission fissures and replicate by division and rarely have apothecia (Knudsen et al. 2021a; Vondrák et al. 2022).

Continuing our study of Acarosporaceae in North America, we describe four new species of *Acarospora* from California and a squamulose *Sarcogyne* from New Mexico and California. We published a key to Acarosporaceae of southwestern United States recently and we explain how each species described in this paper would be inserted into the key (Knudsen et al. 2023a). We publish a table of the verified Acarosporaceae of North America north of Mexico (Table 1). As a contribution to the development of a new checklist of lichens in California, we publish a table of verified species of Acarosporaceae occurring in California (Table 2).

## ﻿Materials and methods

### ﻿Herbarium study

Collections of Acarosporaceae were studied from SBBG and the private herbarium of Kocourková and Knudsen (hb. K&K). The isotype of *Acarosporaobscura* and collections of *A.lapponica* were studied from H and PRM. The morphology of specimens was examined with dissecting microscopes. At 1000× with compound microscopes the anatomy of hand sections was measured in water. The amyloid reaction of the hymenial gel and subhymenium was tested with fresh undiluted IKI (Merck’s Lugol, Sigma-Aldrich 1.09261); see protocol for repeatable results in Knudsen and Kocourková (2018) and Knudsen et al. (2024a). The ascus stain was studied in IKI (Hafellner 1993). Thin-layer chromatography (TLC) in solvents A, B’, C was performed to identify secondary metabolites (Orange et al. 2010). On completion of the study holotypes, isotypes and some paratype material were placed in PRM or SBBG. All specimens collected in Joshua Tree National Park were deposited in SBBG.

### ﻿Imaging

Macrophotographs were taken with the digital camera Olympus DP74 mounted on Olympus SZX 16 stereomicroscope using PROMICRA QuickPHOTO CAMERA 3.3 software and stacked using Olympus DeepFocus 3.5 module for increasing the depth of field. Microphotographs were taken with a digital camera Olympus DP74 mounted on an Olympus BX51 light microscope fitted with Nomarski interference contrast and using PROMICRA QuickPHOTO CAMERA 3.3 software. The figure plates were processed with the module Figure Maker fitted to the same software.

### ﻿DNA extraction, PCR amplification and sequencing

DNA was extracted from 31 dried herbarium specimens (Suppl. material 2) via the Invisorb Spin Plant Mini Kit, according to the manufacturer’s protocol with slight modifications (i.e. eluted in 50 μL of DNA, instead of 100 μL, and incubated in buffer for 15 minutes before final centrifuging). Total extracted DNA was stored at -20 °C. The quality and yield of DNA isolated was checked on a 1% agarose gel and DNA concentration and purity were then measured precisely using a UVS‐99 spectrophotometer (ACTGene). The selected markers for this study were the internal transcribed spacer (ITS; White et al. 1990), the large subunit of the nuclear ribosomal DNA (nrLSU; Vilgalys and Hester 1990), and the small subunit of the mitochondrial ribosomal DNA (mtSSU; Zoller et al. 1999). The ITS, nrLSU, and mtSSU regions were amplified via polymerase chain reaction (PCR).

Each reaction contained 1 μL (20–25 ng) of extracted genomic DNA, 10 μL of 2× MyTaq Red DNA Polymerase (Bioline), 8.2 μL of water, 0.4 μM of forward/ reverse primer (10 μM) for a total reaction volume of 20 μl. Conditions for nrITS, mtSSU rDNA: initial denaturation 95 °C for 5 min, followed by five cycles (95 °C for 33 s, 56 °C for 30 s, and 72 °C for 30 s), then ten cycles (95 °C for 30 s, 54 °C for 30 s, and 72 °C for 30 s), and twenty cycles (95 °C for 30 s, 50 °C for 30 s, and 72 °C for 30 s) with a final extension 72 °C for 15 min. Conditions for the nrLSU: initial denaturation 95 °C for 1 min, followed by five cycles (95 °C for 30 s, 55 °C for 30 s, and 72 °C for 60 s) and finally 30 cycles (95 °C for 30 s, 52 °C for 30 s, and 72 °C for 60 s), with a final extension 72 °C for 10 min. Before sequencing, the PCR products were purified using the enzymatic method ExoSap-ITTM Express Reagent provided by Thermo Fisher (Scientific, Inc.) according to the manufacturer’s protocol. PCR products were run on a 1.0% agarose gel via electrophoresis and stained with ethidium bromide for 20 min. Purified PCR products, water, and forward primer (8 μL in total volume) were sequenced by BIOCEV, Vestec, Czech Republic.

### ﻿Sequence alignment and phylogenetic analysis

The newly sequenced ITS, mtSSU, and nrLSU were aligned with previously published data (see Suppl. material 2) for strain information and accession numbers). Multiple sequence alignment was performed for each gene separately using MUSCLE v3.6 before concatenating them into a super-alignment for phylogenetic analysis (Edgar 2004). The best partition scheme and substitution models (TIM2e+I+G4 for ITS, TPM2+F+I+G4 for mtSSU, and TIMe+I+G4 for nrLSU) were identified using the IQ-TREE version 1.6.12 package. Phylogenetic trees were constructed using MRBAYES 3.2.2 (Ronquist and Huelsenbeck 2003). Input data was formatted for MRBAYES via the FABOX online converter tool to create a MRBAYES input file (http://birc.au.dk/~palle/php/fabox/fasta2mrbayes.php; Villesen 2007).

**Figure 1.**
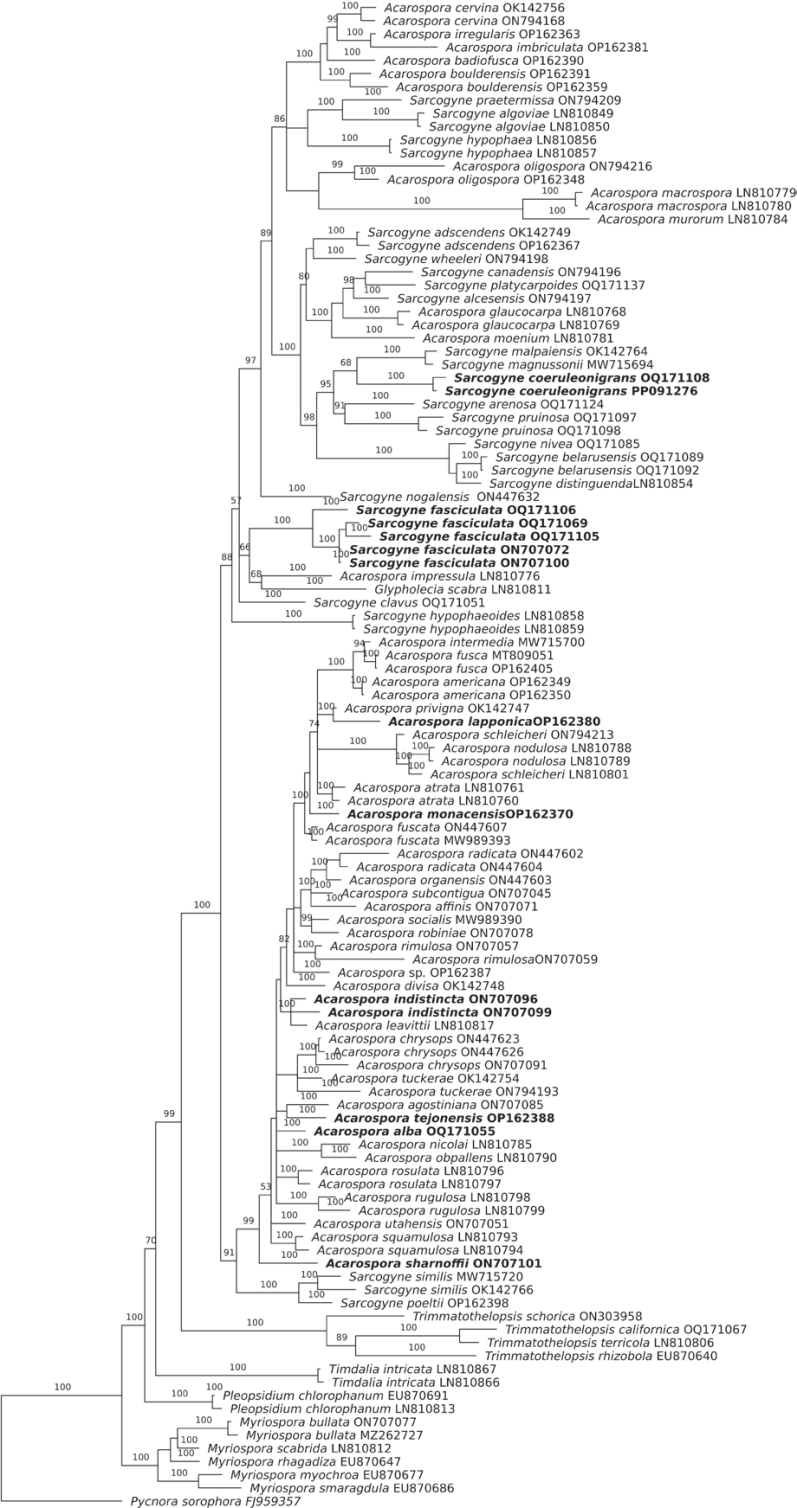
Bayesian inference tree obtained by phylogenetic analysis using a combined data set of ITS, mtSSU, and nLSU sequences of 114 members of Acarosporaceae. Bayesian posterior probability (BPP) is indicated above branches. *Pycnorasorophora* was used as outgroup. The number after the species name represents the GenBank accession number of the ITS sequence.

Sequences of *Pycnorasorophora* were included as an outgroup. Three replicate analyses with four chains each were computed 30,000,000 generations, sampling every 1 000^th^ generation. After this number of runs, the average standard deviation of split frequencies reached a value lower than 0.01, indicating that convergence was reached. The data were analyzed using maximum likelihood (ML) methods too, followed by the reconstruction of the maximum-likelihood tree (Nguyen et al. 2015; Kalyaanamoorthy et al. 2017). Branch supports were assessed using bootstrap approximation (1,000 replicates) for Bayes and ultrafast bootstrap approximation with 1,000 replicates for ML method (Hoang et al. 2017). The concatenated alignment and associated tree files are accessible at Zenodo (https://doi.org/10.5281/zenodo.10700705). The final alignment contained 115 (114 for ingroup) taxa with 1,572 concatenated characters, consisting of 1–437 (ITS), 438–1,031 (mtSSU), and 1,032–1,572 (nrLSU) nucleotide sites. Of these characters, 708 were variable and phylogenetic informative. The maximum likelihood Bayesian tree with bootstrap supports is presented in Fig. 1 and ML tree is presented in Suppl. material 1.

## ﻿Results and discussion

Our current family tree is congruent with previous family trees (Westberg et al. 2015; Knudsen et al. 2020, 2023a). Two clades *Acarospora* and *Sarcogyne* are recovered. Both *Acarospora* and *Sarcogyne* are not monophyletic. Current genus names of species have not been changed anticipating further phylogenetic and genomic analysis and wider taxon sampling.

*Sarcogynefasciculata* K. Knudsen, Kocourk. & Hodková (bold in Fig. 1) in our phylogeny is recovered in a poorly supported clade in *Sarcogyne* sister to *Acarosporaimpressula* and *Glypholeciascabra*. *Glypholecia* is a genus we do not recognize. *Glypholecia* was described based on the morphological concept that compound apothecia were a synapomorphic character in the Acarosporaceae (Ryan 2002). Compound apothecia occur in other species such as *A.lapponica*, *A.privigna* and *Trimmatothelopsisamericana*. *Glypholeciascabra* does not form a monophyletic clade of species with compound apothecia but is recovered in *Sarcogyne*. *Sarcogynefasciculata* does not have compound apothecia. It is among those species recovered in the *Sarcogyne* clade which would have been described as an *Acarospora* because of immersed apothecia using morphological species concepts. *Sarcogynefasciculata* is similar to *S.nogalensis* and both have euamyloid hymenial gel (Knudsen et al. 2023a). *Sarcogynefasciculata* is currently known from the Chihuahuan Desert in New Mexico, and in southern California in the Mojave Desert in Joshua Tree National Park and in the montane lichen flora in the San Bernardino Mountains.

*Acarosporaalba* K. Knudsen, Kocourk. & Hodková (bold in Fig. 1) in our phylogeny is in an isolated position in *Acarospora* clade. *Acarosporaalba* is currently known only from the Mojave Desert lichen flora in Joshua Tree National Park in southern California. It is rare, known from only two collections. The second collection was infected by *Acarosporadestructans*, a common lichenicolous fungus, and sequences were contaminated. The unstratified thallus is common in several *Sarcogyne* species such as *S.magnussonii* and in the rare European species *A.variegata* (Knudsen and Kocourková 2012; Knudsen et al. 2024a).

*Acarosporatejonensis* K. Knudsen & Kocourk (bold in Fig. 1). is currently only known from California in the Tehachapi Mountains, the Carrizo Plain, and on Santa Rosa Island. Only the type collection was sequenced. The two other collections were too small to be sequenced, and sampling beyond taxonomic analysis would be destructive. The sister of *A.tejonensis* is *A.agostiniana* which produces gyrophoric acid and is currently only known from the Chihuahuan Desert in New Mexico (Knudsen et al. 2023a).

*Acarosporaindistincta* K. Knudsen, Kocourk. & Hodková (bold in Fig. 1) in our phylogeny is recovered in a lineage with *Acarosporaleavittii*, a southwestern North American desert species which can produce ascospores small globose to 7×5 µm or larger (Magnusson 1952, as *Sarcogyneoligospora*.; Knudsen et al. 2021b). These sequences of *A.leavittii* from the Mojave Desert were originally misidentified and loaded into GenBank as the Asian species *Sarcogynegyrocarpa* (Knudsen and Kocourková 2009b; Westberg et al. 2015). Based on new sequences of *Sarcogynegyrocarpa* from Asia, the recovered sequences from North America are currently recognized as the North American species *A.leavittii* (Knudsen et al. 2021b). *Acarosporaindistincta* also differs from *A.leavittii* in having an epilithic thallus of squamules with immersed apothecia without carbonized epihymenial accretions vs. an endolithic thallus with black lecideine apothecia with carbonized epihymenial accretions. *Acarosporaindistincta* is currently known from eight collections from the Mojave and Sonoran Desert in Joshua Tree National Park.

*Acarosporasharnoffii* K. Knudsen, Kocourk. & Hodková (bold in Fig. 1) in our phylogeny is in an isolated position. Currently *A.sharnoffii* is only known from the Little San Bernardino and Queen Mountains in Joshua Tree National Park and is part of the Mojave Desert lichen flora but may like *Sarcogynefasciculata* belong also to the montane lichen flora of California. Squamules are covered with abscission fissures, predominately replicating by division, with few apothecia. The species is usually contaminated with lichenicolous fungi, including *Endococcus* and the common desert species *Lichenotheliaconvexa*.

*Sarcogynecoeruleonigricans* (in bold in Fig. 1) is the first of the *Sarcogyne* species on calcareous rock to be described from North America (Knudsen et al. 2023a, c). Several calciphytes were identified as *S.pruinosa* or its synonym *S.regularis* in the misapplication of European names to North America taxa. We do not recognize that *S.pruinosa* occurs in North America until it is proven. Like *S.pruinosa*, *S.coeruleonigrians* has a wide range genetic and anatomical variation in the width of apothecia and the margin, convexity of apothecia, and hymenial height (Knudsen et al. 2023a, c). *Sarcogynecoeruleonigricans* has unstable IKI reactions of hymenial gel from euamyloid to hemiamyloid and is not a useful diagnostic character. It has been reported from the Sonoran Desert in Arizona and from the Chihuahuan Desert in New Mexico, and from northern Mexico. In our phylogeny a collection on caliche in the San Jacinto Mountains along the Sonoran Desert interface is recovered with a collection from the Chihuahuan Desert of New Mexico. Both specimens had euamyloid hymenial gel reactions and both were a more reduced form collected especially in sunny, very dry sites. *Sarcogynecoeruleonigricans* is reported new for California. Though other specimens have not been revised, *S.regularis* was reported previously from the San Jacinto and San Bernardino Mountains (Knudsen et al. 2017).

New sequences for *Acarosporaamericana* from California, a common species in North America, are published for comparison with the new species *A.tejonensis*. The two species are sympatric in central California. *Acarosporaamericana* is most closely related to two common species from Europe, *A.fusca* and *A.intermedia*, and is not closely related to *A.tejonensis*. We have seen no specimens of *A.americana* from Europe. The name is sometimes misapplied in southwestern determinations to taxa with one disc and pruina in the *A.strigata* group (CLH 2023).

*Acarosporalapponica* is recovered in *Acarospora*. It was treated as *Sarcogynelapponica* (Knudsen and Kocourková 2009a). *Acarosporalapponica* is a Holarctic species (Magnusson 1929). In our phylogeny our Czech specimen of *A.lapponica* is sister to *A.privigna* s. lato from the Hartz Mountains in Germany. The outer wall of the apothecia is carbonized, with a hyaline area between the outer wall and parathecium formed from medullary hyphae, sometimes with algae (extending upward from algal layer at base of apothecia). The disc varies from lacking carbonized epihymenial accretions to eventually having one umbo, sometimes with gyrose structures. The holotype occurred on wood. It was recently collected on wood in France (Roux and Poumarat 2023). We recently identified a specimen from Algeria, extending its southern range from France to north Africa (C. Flagey Lichenes Algerienses 132, H!). *Acarosporalapponica* needs molecular sampling across its Holarctic distribution because probably more than one species with similar anatomy is involved.

*Acarosporamonacensis* is recovered in an isolated position in *Acarospora*. It is an apparently rare species known only from the Czech Republic and Germany (Knudsen et al. 2024a). It was recently rescued from synonymy with *A.fuscata* which in our phylogeny is not closely related.

We publish for first time sequences of *Acarosporarimulosa* and *A.subcontigua*, two poorly known yellow species from the Chihuahuan Desert in New Mexico, as well as new sequences of *A.chrysops*, *A.radicata* and *A.socialis*. Not surprisingly, two yellow species that are endemic to the Pacific Coast, *A.socialis* (type locality, Santa Catalina Island) and *A.robiniae* (type locality Santa Cruz Island), are sisters in our phylogeny (Fig. 1).

### ﻿Taxonomy

#### ﻿ Acarospora alba

Taxon classificationFungiAcarosporalesAcarosporaceae

K. Knudsen, Kocourk. & Hodková sp. nov.

F4ACFFE2-03BD-51B4-8215-E4147A100F9E

MB853899

Fig. 2

##### Type.

U.S.A. • California: Riverside Co., Joshua Tree National Park, Mojave Desert, Sheep’s Pass, at base of Ryan Mountain, on gentle west-facing slope covered with small rocks and pebbles of granite and gneiss, 34.001, -116.1268, alt. 1369 m, on granite, 20 Dec 2010, K. Knudsen 13222 (holotype-SBBG).

**Figure 2.**
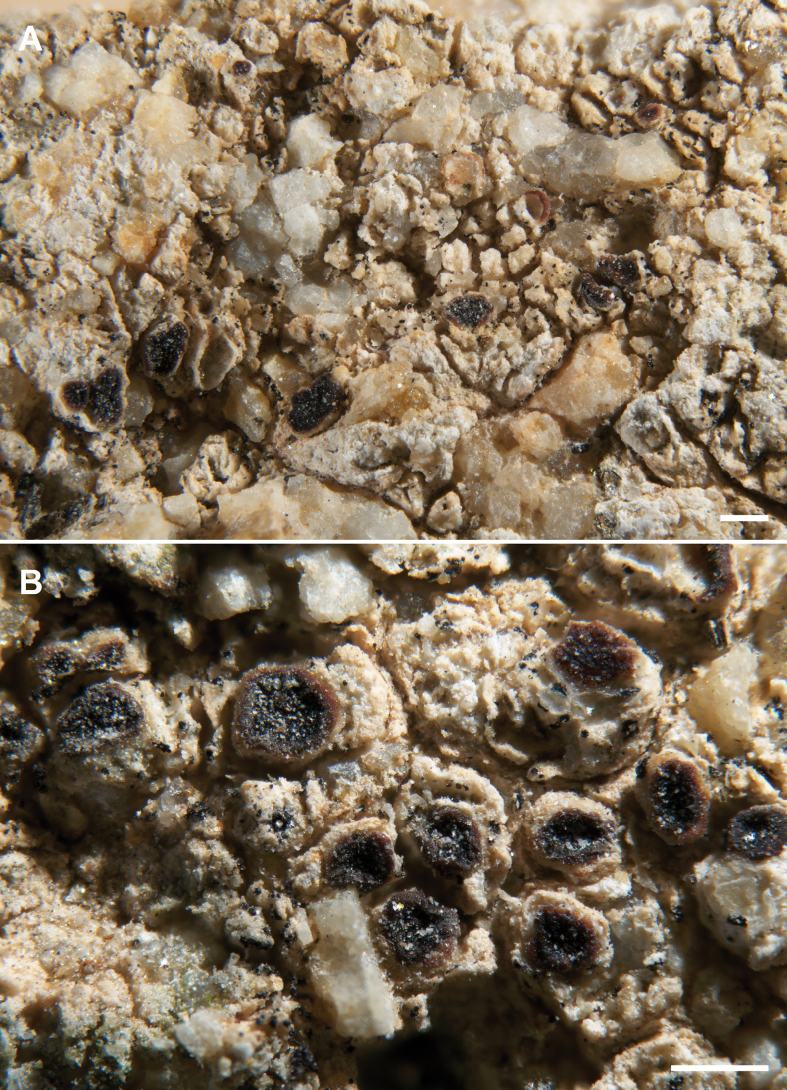
*Acarosporaalba*, Knudsen 13181 Isotype **A** chasmolithic thallus and young apotecia **B** apothecia one per areole with thin white margin (Holotype). Scale bars: 500 μm (**A, B**).

##### Diagnosis.

Similar in having a non-stratified thallus like the possibly extinct central European species *Acarosporavariegata* but differing in not producing gyrophoric acid, not developing a distinct cortical layer which is poorly developed in *A.variegata*, and having an opaque white upper surface, not translucent in water.

##### Etymology.

Named for the white non-translucent upper surface of the unstratified thallus.

##### Description.

Thallus growing in the upper layer of substrate, endolithic to epilithic, covering 1–3 cm in width, an unstratified matrix of gelatinized intricate to anticlinal hyphae, 1–4 µm wide, intermixed with substrate crystal, with thin cracks splitting the upper layer into areoles irregular in shape, 0.3–1.0 mm wide, 200–400 µm thick. Upper surface ecorticate, white, epruinose, sometimes with small pale patches of reddish-brown pigment observed in thin sections at 1000×, not translucent when wet. Algae scattered or in thin clusters, algal cells 7–16 µm wide, not forming a continuous algal layer. Apothecia 0.1–0.5 mm wide, usually one per areole, oval to irregular in shape, disc black when dry, dull reddish brown when wet or not changing color, epruinose to lightly pruinose, rugulose or smooth, immersed and even with thallus surface or emergent and elevated above the thallus surface, with a thin white thalline margin 20–50 µm wide. Parathecium 10–40 µm wide, hyphae 2 µm wide, sometimes visible as parathecial ring around apothecial disc same color as epihymenium. Hymenium (100–)120–150 µm tall, cupular, usually tallest in center, epihymenium ca. 10 µm tall, reddish-brown, paraphyses mostly 1 µm wide, not branching, apices unexpanded, hymenial gel IKI+ blue to red, hemiamyloid. Asci 80–90 × 22–26 µm, clavate, ascospores mostly 3–4 × 2 µm, ellipsoid, several hundred per ascus (n = 20). Subhymenium 20–25 µm tall, IKI+ blue, euamyloid. Hypothecium Y-shaped, the central axis of hyphae extending down in a bundle, the arms 15–30 µm thick embracing the V-shaped subhymenium and continuous with parathecium. Pycnidia not observed. Chemistry: not producing secondary metabolites.

##### Habitat and distribution.

Known only from two locations on granite in full sun in the Mojave Desert in Joshua Tree National Park at approximately the same elevation of 1347–1369 m. The two specimens were collected about ten miles from each other. The sequences of the paratype were contaminated by a common parasitic lichenicolous fungus in Joshua Tree National Park, *Acarosporadestructans*, though the parasite had not produced apothecia, and the thallus of *A.alba* showed no signs of contamination (Knudsen et al. 2022a). Sequences were only obtained from the holotype. Based on extensive collecting in Joshua Tree National Park since 2005 by Knudsen and Kocourková, the species is considered rare (Knudsen et al. 2013; Knudsen and Kocourková 2023).

##### Additional specimen examined.

U.S.A. • California, Riverside Co., Joshua Tree National Park, Mojave Desert, off trail between Skull Rock and Jumbo Rocks, 33.9958, -116.0653, alt. 1347 m, on granite, 19 Dec 2010, K. Knudsen 13181 (SBBG).

##### Notes.

The white pigmentation of the thallus, like the white layer of pruina on *Acarosporapeltastica*, increases surface albedo to protect the algal layer in the intense sunlight of the desert. *Acarosporaalba* has a non-translucent white surface when wet while the white layer of *A.peltastica* is translucent when wet, the brown from a pigmented lower layer visible through the transparent pruina and epicortex. In our current key of Acarosporaceae of southwestern North America *A.alba* is recovered in section 8, couplet 8, with *A.arenacea* and *A.bolleana*, both with non-translucent white surfaces. *Acarosporaarenacea* has euamyloid hymenial gel and thalli with deeply cross-hatched surfaces and ultimately produces elevated lecideine apothecia that are reddish-brown or black with carbonized epihymenial accretions (Magnusson 1952; Knudsen et al. 2023a). *Acarosporabolleana* has hemiamyloid hymenial gel like *A.alba* but differs in having a thallus of scattered white areoles becoming solitary lecideine apothecia. while *A.alba* has a continuous unstratified white thallus with immersed or emergent black apothecia.

#### ﻿ Acarospora indistincta

Taxon classificationFungiAcarosporalesAcarosporaceae

K. Knudsen, Hodková & Kocourk. sp. nov.

0FC4DD0D-2BF1-539F-8465-FA5F25D9C0E3

MB853900

Fig. 3

##### Type.

U.S.A. • California, Riverside Co., Joshua Tree National Park, Mojave Desert, common on lower north slope of Malapai Hill, 33.9431, -116.0875, alt. 1190 m, on basalt, 5 Dec 2012, K. Knudsen 12772 (holotype, isotype-SBBG).

**Figure 3.**
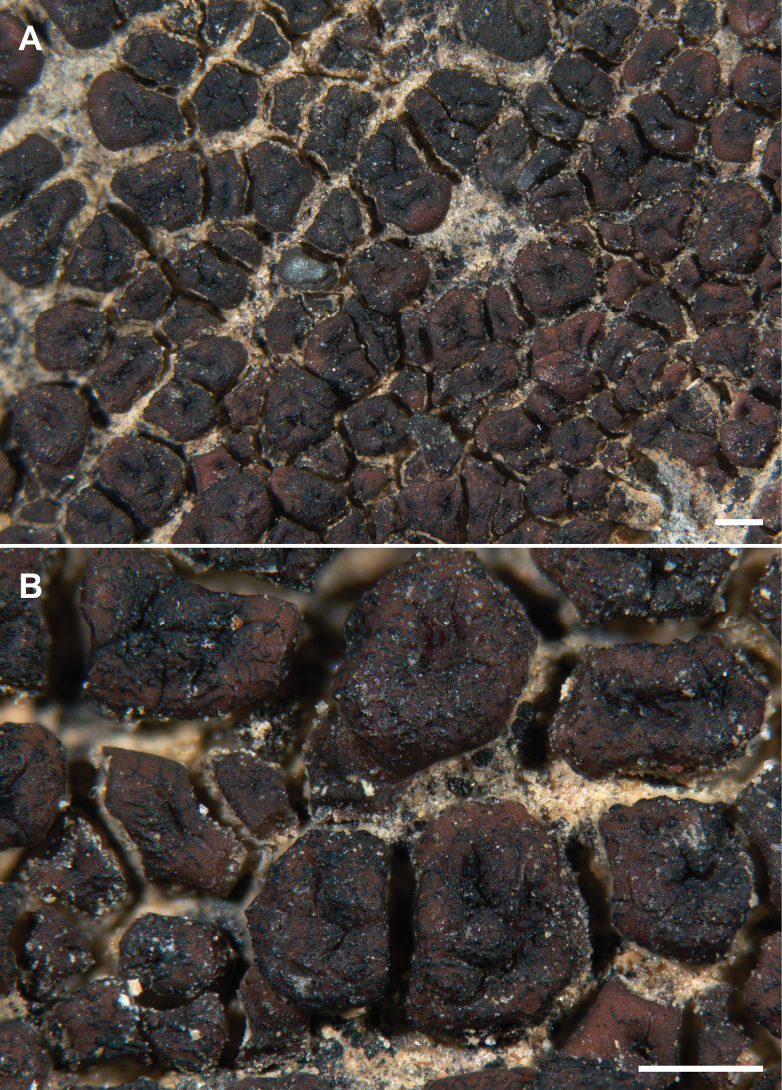
*Acarosporaindistincta* Knudsen 12772 Isotype **A** habit of the thallus of convex dispersed squamules with immersed apothecia **B** detail of squamules (infected with *Lichenostigma* sp.) Scale bars: 500 μm (**A, B**).

##### Diagnosis.

Similar to *Acarosporaveronensis* but differing with a thicker cortex (50–)90–100 vs. 10–20(–30) µm and in being squamulose.

##### Etymology.

Named for its lack of an appealing and distinctive phenotype. Many brown *Acarospora* look similar, especially ones like *A.indistincta* in the morphological *veronensis* group having one immersed apothecium per brown areole or squamule.

##### Description.

Hypothallus endosubstratal, no algae observed. Thallus of convex dispersed squamules, 0.3–1 mm wide, 0.4–0.7 mm thick, with rounded edges, sometimes irregular in shape, usually not replicating by division, covering areas of several centimeters. Upper surface brown, shiny or dull, rarely pruinose. Lower surface white. Epicortex 10–40 µm thick. Cortex (60–)90–100 µm thick, of mostly round cells 2–5 µm wide, upper layer red brown, 10 µm thick, lower layer hyaline. Algal layer 90–150 µm thick, even, dense, continuous below apothecia, not interrupted by hyphal bundles, algal cells 10–15 µm wide. Medulla 180–250 µm thick, of anticlinal hyphae thin-walled and 2 µm thick, continuous with the stipe. The majority of squamules are sterile. Usually one apothecium per areole, immersed, below thallus level, punctiform, expanding up to 0.4 mm wide, disc dark brown, epruinose, sometimes becoming slightly elevated in thalline margin. Parathecium indistinct or 10 µm wide of narrow hyphae 1 µm wide. Hymenium mostly 90–100 µm tall, epihymenium 10–20 µm tall, red-brown, surface uneven, paraphyses 1.0–1.5 µm wide, hymenial gel IKI+ blue turning red, hemiamyloid. Asci clavate 50–60 × 15–21 µm, ascospores several hundred, thin ellipsoid, 3–4 × 1–1.5 µm (n = 20). Subhymenium 40–50 µm tall, IKI+ blue, euamyloid. Hypothecium 20–30 µm tall, IKI-. Pycnidia not observed. Chemistry: not producing secondary metabolites.

##### Habitat and distribution.

*Acarosporaindistincta* is currently only known from Joshua Tree National Park in the Mojave Desert and Sonoran Desert on granite and basalt in full sun.

##### Additional specimens examined.

U.S.A. • California, Riverside Co., Joshua Tree National Park, Mojave Desert, Little San Bernardino Mountains, Eureka Peak, north of summit in canyon, 34.0344, -116.3477, alt. 1591 m, on granite, 22 Feb 2006, K. Knudsen et al. 5249 (SBBG); • Eureka Peak, below summit, 34.0317, -116.3487, alt. 1670 m, on granite on steep north-facing slope, 28 March 2023, J. Kocourková 11125 & K. Knudsen (SBBG); • Malapai Hill, 33.9375, -116.0843, alt. 1165 m, on basalt rubble at base of hill, 5 Dec 2010, K. Knudsen 12637.2 (SBBG); • Sonoran Desert, north slope of Cottonwood Mountains, Pinkham Canyon 33.7787, -115.9317, alt. 970 m, on granite, 8 Dec 2010, K. Knudsen 12897.1 (SBBG); • San Bernardino Co., Joshua Tree National Park, Mojave Desert, Queen Mountain, 34.0523, -116.1031, alt. 1636 m, rare on granite, 5 Oct 2012., K. Knudsen 13721 & M. Harding (SBBG); • just outside Joshua Tree National Park, off Covington Flats Rd., along road to radio tower, 34.0766, -116.3497, alt. 1219 m, on granite, 9 Apr 2006, K. Knudsen 5782 (PH, SBBG).

##### Notes.

In our current key of Acarosporaceae of southwestern North America, *Acarosporaindistincta* is recovered in section 8, couplet 11, with squamules epruinose, differing from *Trimmatothelopsisoreophila* in having a shorter hymenium 90–100 vs. (130–)170–220(–250) μm high (Knudsen et al. 2023a).

#### ﻿ Acarospora sharnoffii

Taxon classificationFungiAcarosporalesAcarosporaceae

K. Knudsen, Hodková & Kocourk. sp. nov.

8C2D862B-0506-5C3E-A1F0-8FF725AA6418

MB853901

Fig. 4

##### Type.

U.S.A. • California, Riverside Co., Joshua Tree National Park, Mojave Desert, Little San Bernardino Mountains, Eureka Peak, below and west of parking area, 34.0197, -116.3630, 1650 m, on granite, 16 Jan 2012, S. Sharnoff 4107 (holotype-SBBG).

**Figure 4.**
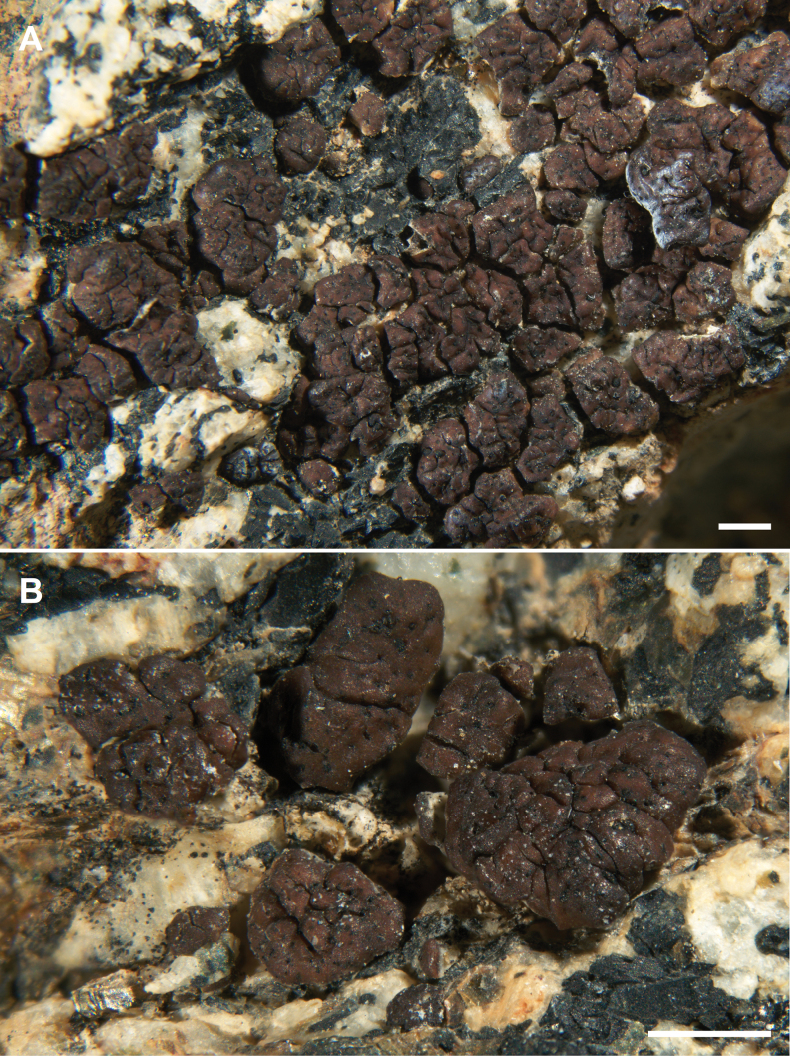
*Acarosporasharnoffii* Kocourková 11113 Paratype **A** habit of the sterile thallus (infected with *Endococcus* sp.) **B** squamules with abscission fissures. Scale bars: 1 mm (**A, B**).

##### Diagnosis.

Similar to *Acarosporaapplanata* but differing in being squamulose.

##### Etymology.

Named after Stephen Sharnoff, lichen photographer, who produced the classic book A Field Guide to California Lichens (Sharnoff 2014). He collected the first specimen on Eureka Peak in Joshua Tree National Park. A picture of the holotype of *Acarosporasharnoffii* as *A.obnubila* is in his book.

##### Description.

Hypothallus endosubstratal, no algae observed. Thallus of squamules dispersed to contiguous, 0.5–2.0 mm wide, with stipe raised distinctly above substrate, 0.3–0.45 mm thick, with an uneven and irregular topography, replicating by division. Upper surface shiny brown, epruinose or pruinose, with abundant abscission fissures. Lower surface ecorticate, white or brown. Epicortex continuous, 10–40 µm thick. Cortex 20–60 µm thick, upper layer brown 8–12 µm thick, lower layer hyaline, of disarticulated anticlinal hyphae, cells round to irregular, 2–5 µm wide. Algal layer 70–100 µm thick, upper surface even to uneven, continuous below apothecia, algal cells mostly 10–12 µm wide. Medulla 100–200 µm thick, hyphae thin-walled, mostly 4 µm wide, continuous with stipe, becoming periclinal along lower surface. Apothecia rare, usually one per squamule in center, deeply immersed, disc blackish when dry, brownish when wet, 0.3–0.5 mm wide, epruinose, rough, rarely disc expanded to 1 mm wide, reducing the squamule to thalline margin. Parathecium of narrow hyphae 1 µm wide, expanding to ca. 20 µm wide around disc, merging into the cortex. Epihymenium 8–12 µm tall, surface uneven, brown. Hymenium 70–90(–110) µm tall, epihymenium 10 µm tall blackish brown, paraphyses 1.0–1.5 µm wide, apices barely expanded in gel caps, hymenial gel IKI+ blue to red, hemiamyloid. Asci cylindrical, 50–80 × 10–15 µm wide, ascospores usually small, 2.0–4.0 × 1.0–1.5 µm, often with two oil drops (n = 20). Subhymenium 25–45 µm tall, IKI+ blue, euamyloid. Hypothecium 10 µm thick. Pycnidia not observed. Chemistry: not producing secondary metabolites.

##### Habitat and distribution.

The species is currently only known from the Mojave Desert in Joshua Tree National Park, on Eureka Peak in Little San Bernardino Mountains and on Queen Mountain, on granite from 1627–1670 m. Both these mountain ranges have not been fully explored for lichens. Like *Sarcogynefasciculata* it could also be a montane species and could possibly be collected in the San Jacinto or San Bernardino Mountains in Southern California. Since specimens are predominately sterile it may have been collected but never identified.

##### Additional specimens examined.

U.S.A. • California, Riverside Co., Joshua Tree National Park, Mojave Desert, Little San Bernardino Mountains, Eureka Peak, below summit, on steep north-facing slope, 34.0197, -116.3630, alt. 1670 m, on granite, contaminated with *Endococcus*, 28 March 2023, J. Kocourková 11113 & K. Knudsen (SBBG); • San Bernardino Co., Joshua Tree National Park, Mojave Desert, Queen Mountain, 34.0527, -116.1026, alt. 1627 m, on granite rock next to drainage, epruinose, contaminated with *Lichenotheliaconvexa*, 5 Oct. 2005, K. Knudsen 13749.1 & M. Harding (SBBG) .

##### Notes.

The specimen of *Acarosporasharnoffii* from Queen Mountain was poor compared to the two collections from Eureka Peak. It was infected with *Lichenotheliaconvexa* (syn. *Lichenostigmasaxicola*), an abundant lichenicolous fungus in Joshua Tree National Park (Knudsen and Kocourková 2011). The Kocourková collection from Eureka Peak was also contaminated by lichenicolous fungi.

*Acarosporaapplanata* and *A.fissurata* from New Mexico, *A.fissa* from Czech Republic, and *A.scrobiculata* from Greenland are also cross hatched with abscission fissures, replicating by division, and rarely producing apothecia (Magnusson 1935; Knudsen et al. 2021a, 2023a; Vondrák et al. 2022). In our current key to Acarosporaceae of southwestern North America, *Acarosporasharnoffii* is recovered in squamules on non-calcareous rock, Section 8, couplet 11 with *A.superfusa* from which it differs in being heavily fissured and usually sterile and epruinose (Knudsen et al. 2023a). Both species are sympatric at the type locality on Eureka Peak (Knudsen and Kocourková 2023).

#### ﻿ Acarospora tejonensis

Taxon classificationFungiAcarosporalesAcarosporaceae

K.Knudsen & Kocourk. sp. nov.

CCD32E78-A3B6-58F7-A0B5-E1EE12CA072B

MB853902

Fig. 5

##### Type.

U.S.A. • Kern Co., Tehachapi Mountains, Tejon Ranch, Martinez Ridge, fir and oak forest, 34.9352, -118.6477, alt. 1738 m, on granite, 19 April 2016, K. Knudsen 18838 (SBBG-holotype, isotypes).

**Figure 5.**
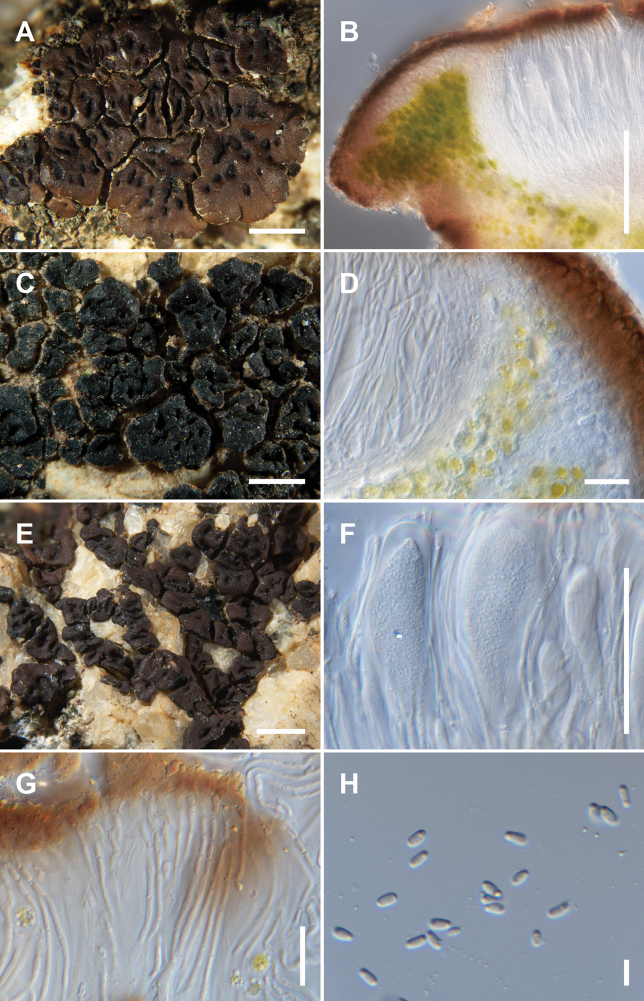
*Acarosporatejonensis* Knudsen 18838 Holotype (**A–C, F, H**), Knudsen 18838 Isotype (**D, E, G**) **A** habit of the thallus with prolongated marginal areoles **B** vertical section through apothecium and margin of areole **C** variability of the thallus, black areoles with immersed apothecia sometimes reduced to a prominent margin **D** vertical section through apothecium showing parathecium with narrow hyphae **E** thallus of areoles lining crevices **F** young asci showing tholus **G** paraphyses slightly widened at tips, terminal cells orange-brown **H** ascospores. Scale bars: 1 mm (**A, C, E**); 100 µm (**B**); 20 µm (**D, G**); 50 µm (**F**); 5 µm (**H**).

##### Diagnosis.

Similar to *A.veronensis* but with a thicker cortex, 30–50(–70) vs.15–30 µm and a usually higher hymenium (100–)120(–150) vs. 80–90(–100) µm.

##### Etymology.

Named after the type locality in Tejon Ranch in Tehachapi Mountains.

##### Description.

Hypothallus endosubstratal, no algae observed. Thallus areolate, areoles 0.2–1.1 mm wide, 150–270 µm thick, angular, contiguous to dispersed, marginal areoles can be prolongated and lobate, becoming elevated by a mycelial base, replicating by division, covering areas up to 4 cm or more. Upper surface light to dark brown, sometimes partly black, epruinose, rugulose to smooth. Lower surface white. Epicortex lacking. Cortex 30–50(–70) µm thick, upper layer dark brown, 10–30 µm thick, lower layer hyaline, hyphae usually disarticulated, cells round to irregular, mostly small, 1–3 µm wide, rarely up to 5 µm wide. Algal layer up to 100 µm thick, uninterrupted, algal cells 8–12 µm wide, continuous below apothecia. Medulla white, 0.2–0.7 mm thick, hyphae obscure in water, intricate and gelatinized, mostly 1 µm wide. Apothecia immersed, usually one per areole, sometimes 2–5, sometimes with areole reduced to a prominent margin, the disc dark, epruinose, same color as thallus, 0.1–0.3 mm wide, rarely to 0.5 mm wide. Parathecium indistinct to 20 µm wide of narrow hyphae 1 µm wide, merging with the cortex. Hymenium cupular, (100–)120(–150) µm tall, epihymenium dark reddish brown, 10 µm tall, paraphyses 1–2 µm wide, apices unexpanded or slightly widened in terminal reddish brown gel cap, hymenial gel IKI+ reddish orange, hemiamyloid. Asci 90–120 × 10–20 µm, cylindrical to clavate, ascospore several hundred per asci, small, thin ellipsoid, 3–4 × 1 µm (n = 20). Subhymenium 20–40 µm tall, IKI+ blue. Hypothecium indistinct to 10 µm thick. Pycnidia not observed.Chemistry: not producing secondary metabolites.

##### Habitat and distribution.

U.S.A., California. on Santa Rosa Island (Channel Island National Park), in Carrizo Plain National Monument, and in the Tehachapi Mountains, from 198–1738 m, on sandstone and siliceous rock in full sun.

##### Selected specimens examined.

U.S.A. • California, Santa Barbara Co., Santa Rosa Island, South Point, 33.8950, -120.1159, alt. 183 m, on sandstone growing with *A.socialis*, 14 June 2009, K. Knudsen 11422 (SBBG); • San Luis Obispo Co., Carrizo Plain National Monument, canyon south of Hurricane Road, but near road, Elkhorn Hill, north-facing slope, 35.2074, -119.7026, 838 m, on siliceous rock, 28 March 2016, R. Rosentreter 19464 (SBBG).

##### Notes.

In our key to southwestern North America Acarosporaceae (Knudsen et al. 2023a) *Acarosporatejonensis* is recovered in Section 8 couplet 12, areoles with an average hymenium height of 120 μm in couplet 13 with *Myriosporahassei* and *Acarosporaworthingtoniana*. *Acarosporatejonensis* differs from *M.hassei* in not having an interrupted algal layer or a hymenium up to 200 μm high. It differs from *A.worthingtoniana* in having a thicker cortex [30–50(–70) vs. ca. 20 μm], a narrower parathecium indistinct to 20 μm vs. 40–100 μm wide, and in not having abundant apothecia in each areole.

Because *Acarosporatejonensis* usually has one apothecium per small areole without producing secondary metabolites, it belongs to the morphological *A.veronensis* group. It may be in California collections misidentified as *A.veronensis* if the hymenium and cortex is not measured or as *A.americana* if the parathecium is not measured. *Acarosporatejonensis* differs from *A.veronensis* especially in the having a higher hymenium (100–150 vs. 60–100 µm) and thicker cortex [30–50(–70) vs. 15–30 μm]. *Acarosporatejonensis* differs from *A.americana* in having a narrower parathecium (Knudsen 2021). The two species are sympatric in California. *Acarosporatenebrica* has similar anatomical measurement as *A.tejonensis* except apices of the parathecial hypae widen up to 3 μm. *Acarosporatenebrica* occurs in southwestern Texas and New Mexico while *A.tejonensis* is known only from central California. Magnusson (1956) reported the North African species *Acarosporaobscura* as occurring in California. “Whether the identity between the American and the African specimens is complete is not easy to state owing to the smallness of areoles” (Magnusson 1929). We examined an isotype of *A.obscura* from H. *Acarosporaobscura* has flat smooth brownish-black areoles less than 0.5 mm wide, cortex less than 30 µm thick, broadly attached with a black underside, punctiform immersed apothecia 0.1–0.3 mm wide, parathecium indistinct to 15 µm wide, with a low hymenium less than 100 µm. We have seen no specimens of *A.obscura* from California. *Acarosporatejonensis* can have small dark brownish-black areoles like *A.obscura* but differs in the thicker cortex 30–50(–70) µm, a thickening mycelial base, a higher hymenium usually 120 µm tall. The specimen of *A.tejonensis* from Santa Rosa Island has areoles both small and all black. It is probable Magnusson identified a small blackish specimen of *A.tejonensis* as *A.obscura*.

#### ﻿ Sarcogyne fasciculata

Taxon classificationFungiAcarosporalesAcarosporaceae

K.Knudsen, Kocourk. & Hodková sp. nov.

1A167F0E-BCBE-566B-B3DB-2E975B31CF99

MB853903

Fig. 6

##### Type.

U.S.A. • New Mexico, Lincoln Co., Chihuahuan Desert, Tularosa basin, Oscura, near Road 54, 33.4863, -106.0925, alt. 1475 m, SW-NE oriented crest above the valley, southernmost hill, on northwest facing slope, on acid sandstone outcrop, 17 March 2022, J. Kocourková 10863 (PRM, holotype).

**Figure 6.**
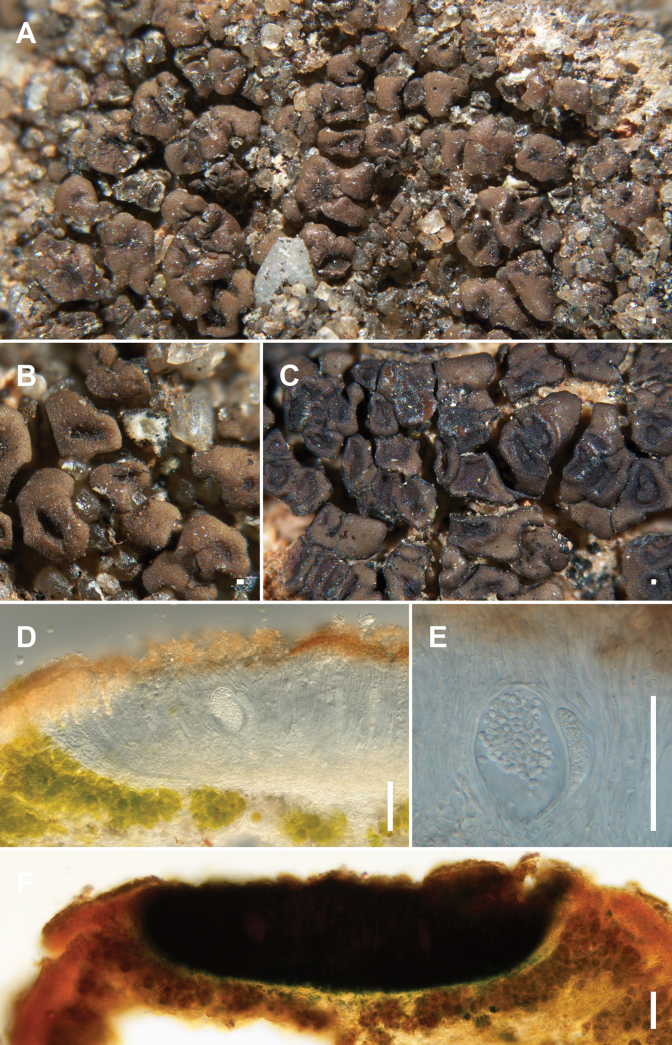
*Sarcogynefasciculata* Kocourková 10863 Holotype (**A, B, D–F**), Kocourková 10971 Paratype (**C**) **A** habit of the thallus **B** detail of stipitate squamules with immersed apothecia **C** apothecia with reduced thallus squamules to thalline margin **D** vertical section of apothecium with developed parathecium **E** young ascus with ascospores **F** IKI+ amyloid reaction of hymenium and subhymenium. Scale bars: 1 mm (**A, C**); 500 μm (**B**); 100 µm (**D, F**), 50 µm (**E**).

##### Diagnosis.

Similar to *Sarcogynenogalensis* but becoming squamulose.

##### Etymology.

Based on the species forming distinctive fascicles of squamules with interconnected stipes while in the process of splitting apart when replicating by division.

##### Description.

Hypothallus endosubstratal, no algae observed. Thallus of squamules or subsquamulose areoles, 0.2–0.5(–1.0–2.0) mm wide, 250–500 μm thick, with stipe 50–200 μm high, forming dispersed to contiguous colonies up to 3×2 cm, sometimes imbricate, with an uneven `topography, replicating by division. Upper surface light or dark brown, rarely shiny, epruinose, the lobes curling downward around the stipe, smooth or with abscission fissures when beginning to replicate by division, forming fasciculate structures of interconnected stipes of squamules during replication. Lower surface pale white sometimes with an undertone of pale brown, corticate with periclinal hyphae, hyaline, up to 20 μm thick. Epicortex, uneven, ca 10 μm thick. Cortex 20–40(–60) μm thick, upper layer ca. 10 μm thick of dark brown round cells, lower layer hyaline of round cells or ellipsoid cells 2–3 × 1.5–2.0 μm. Algal layer 70–120 μm thick, dense, uninterrupted, continuous below apothecia. Medulla obscure, white to pinkish-brown, 100–200 μm thick, hyphae continuous with stipe, 2–4 μm wide, thin-walled, sometimes disarticulated cells expanded or irregular, 4–8 μm wide. Apothecia immersed, darker brown than thallus, in San Bernardino Mountains specimens black, often concave, 0.1–0.6 mm wide, epruinose, occasionally looking pseudolecanorine with squamule reduced to thalline margin, sometimes apothecia in raised parathecial margin. Parathecium up to 60 μm wide, hyphae 2–3 μm wide with apices in brown pigment caps to 5 μm wide, merging with cortex. Hymenium 60–80(–120) μm tall, highest in center, epihymenium 10–15 μm tall, light brown, paraphyses 1.5–2.5 μm wide, apices unexpanded in brown gel caps 2–4 μm wide with upper black pigment line, hymenial gel IKI+ dark blue bleeding into parathecium and hypothecium. Asci (40–)50–75 × 10–20 μm, narrowly cylindrical to inflated clavate, ascospores, 2–4 × 1.5–2.5 µm (n = 20). Subhymenium 20–40 µm tall, IKI+ blue. Hypothecium 10–30 µm tall, hyphae 2 µm wide. No pycnidia observed. Chemistry: not producing secondary metabolites.

##### Habitat and distribution.

*Sarcogynefasciculata* occurs in the Chihuahuan Desert in New Mexico on HCl- sandstone from 1475–1616 m and in southern California on granite in the Little San Bernardino Mountains and the San Bernardino Mountains at elevations 1650–2167 m.

##### Selected specimens examined.

U.S.A. • California, Riverside Co., Joshua Tree National Park, Mojave Desert, Little San Bernardino Mountains, Eureka Peak, E and West of the summit, 34.0132, -116.3502, alt. 1675 m, abundant on granite, 22 Feb 2006, K. Knudsen 5212 (SBBG); • San Bernardino Co., San Bernardino Mountains, conifer forest, above dirt road to Fish Creek, 34.1483, -116.7720, alt. 2167 m, common on granite boulder, 23 Nov 2014, K. Knudsen 17163 (BRY-C, SBBG). • New Mexico, Lincoln Co., Chihuahuan Desert, Carrizozo, Valley of Fires Recreational Area, Malpais Lava Flow, 33.8300, -105.9264, alt. 1616 m, on northwest-facing slope above lava flow, on sandstone pebble, 18 March 2022, J. Kocourková 10848 (hb. K&K), • 33.3503, -105.9227 alt. 1615 m, on northwest-facing slope above lava flow, on sandstone outcrop in full sun, 22 March 2020, J. Kocourková 10974, 10971, 10930 (hb. K&K, SBBG).

##### Notes.

*Sarcogynefasciculata* was first reported from the San Bernardino Mountains as immature *S.squamulosa* (Knudsen and McCune 2013; Knudsen et al. 2017). It was assumed in this population the typical apothecia of *S.squamulosa* had not formed yet. *Sarcogynefasciculata* differs from *S.squamulosa* in not forming elevated brown lecideine apothecia with a parathecium expanded up to 80 µm to form a margin. The apothecia of *S.fasciculata* remain immersed in the squamule or areole and rarely expand reducing the areole or squamule to a thalline margin.

In our current key of Acarosporaceae of southwestern North America *Sarcogynefasciculata* is recovered in Section 8, and couplet 9 brown species with euamyloid hymenial gel with *S.nogalensis* (Knudsen et al. 2023a). Young specimens of *S.fasciculata* that are subsquamulose may be confused with *S.nogalensis*. But *Sarcogynenogalensis* remains areolate and does not become stipitate as well as genetically not being closely related (Fig. 1; Knudsen et al. 2023a).

## ﻿Conclusion

One hundred and twenty species of Acarosporaceae in North America north of Mexico was recently reported (Knudsen et al. 2023a). In revising the diversity of the family in North America, based on current research, we excluded four species. *Acarosporaprivigna* (syn. *Polysporinasimplex*) and *Sarcogynepruinosa* (syn. *S.regularis*) are not proven to occur in North America (Knudsen et al. 2023a, c). We excluded the dubious inclusion in the family of *Eiglera* (1 species). The occurrence of *Myriosporascabrida* in North America needs verification after the discovery that recent reports by Knudsen were the new species *Trimmatothelopsiscalifornica* (Knudsen 2007; Knudsen et al. 2023a). The report of *Myriosporascabrida* for Alaska is based on a 1956 identification by Thomson when the species concept was uncertain (Spribille et al. 2023).

We accepted *A.rugulosa* (=*A.montana*) as occurring in North America based on a determination by Westberg (Dillman et al. 2012). Other new species are *Acarosporahysgina* (Westberg et al. 2024), four new species of *Trimmatothelopsis* (Knudsen et al. 2023a, 2024b), *Sarcogyneadscendens* (Knudsen et al. 2023d), and the five species described in this paper. We report 127 verified species of Acarosporaceae for North America (Table 1).

**Table 1.** One hundred and twenty-seven species of Acarosporaceae in North America north of Mexico.

*Acarosporaaffinis*	*Acarosporanashii*	*Myriosporatangerina*
*Acarosporaagostiniana*	*Acarosporanicolai*	*Pleopsidiumchlorophanum*
*Acarosporaalba*	*Acarosporanodulosa*	*Pleopsidiumflavum*
*Acarosporaamabilis*	*Acarosporanovomexicana*	*Sarcogyneadscendens*
*Acarosporaamericana*	*Acarosporaobpallens*	*Sarcogynealbothallina*
*Acarosporaapplanata*	*Acarosporaoligospora*	*Sarcogynealcesensis*
*Acarosporaarenacea*	*Acarosporaorcuttii*	*Sarcogynearenosa*
*Acarosporabadiofusca*	*Acarosporaobpallens*	*Sarcogynebernardinensis*
*Acarosporabolleana*	*Acarosporaorganensis*	*Sarcogynebrouardiana*
*Acarosporaboulderensis*	*Acarosporapeltastica*	*Sarcogynecalifornica*
*Acarosporabrattiae*	*Acarosporapiedmontensis*	*Sarcogynecanadensis*
*Acarosporabrodoana*	*Acarosporaradicata*	*Sarcogyneclavus*
*Acarosporabrouardii*	*Acarosporarosulata*	*Sarcogynecoeruleonigricans*
*Acarosporabrucei*	*Acarosporarouxii*	*Sarcogyneconvexa*
*Acarosporabullata*	*Acarosporarugulosa*	*Sarcogynecrustacea*
*Acarosporacalcarea*	*Acarosporaryanii*	*Sarcogynedakotensis*
*Acarosporacarnegiei*	*Acarosporaschleicheri*	*Sarcogynedesolata*
*Acarosporachrysops*	*Acarosporascottii*	*Sarcogynefasciculata*
*Acarosporaclauzadeana*	*Acarosporasharnoffii*	*Sarcogynehypophaea*
*Acarosporacoloradiana*	*Acarosporasinopica*	*Sarcogynehypophaeoides*
*Acarosporacontigua*	*Acarosporasocialis*	*Sarcogyneintegra*
*Acarosporadestructans*	*Acarosporasubcontigua*	*Sarcogynemagnussonii*
*Acarosporaelevata*	*Acarosporasquamulosa*	*Sarcogynemalpaiensis*
*Acarosporaepilutescens*	*Acarosporastapfiana*	*Sarcogynemitziae*
*Acarosporaerratica*	*Acarosporasuccedens*	*Sarcogynenogalensis*
*Acarosporaerythrophora*	*Acarosporasuperfusa*	*Sarcogynenovomexicana*
*Acarosporafissurata*	*Acarosporatejonensis*	*Sarcogyneparadoxa*
*Acarosporafuscata*	*Acarosporatenebrica*	*Sarcogyneplicata*
*Acarosporafuscescens*	*Acarosporathamnina*	*Sarcogynepusilla*
*Acarosporaheufleriana*	*Acarosporathelococcoides*	*Sarcogynesimilis*
*Acarosporahysgina*	*Acarosporatintickiana*	*Sarcogynesquamulosa*
*Acarosporaimpressula*	*Acarosporatoensbergii*	*Sarcogyneurceolata*
*Acarosporaindistincta*	*Acarosporatuckerae*	*Sarcogynewheeleri*
*Acarosporainterjecta*	*Acarosporautahensis*	*Trimmatothelopsisamericana*
Acarosporainterpositavar.nitidella	*Acarosporaveronensis*	*Trimmatothelopsiscalifornica*
*Acarosporajanae*	*Acarosporaworthingtoniana*	*Trimmatothelopsisdispersa*
*Acarosporalapponica*	*Glypholeciascabra*	*Trimmatothelopsisnovomexicana*
*Acarosporaleavittii*	*Myriosporadilatata*	*Trimmatothelopsisoreophila*
*Acarosporalendermeri*	*Myriosporahassei*	*Trimmatothelopsisschorica*
*Acarosporamaccarthyi*	*Myriosporamolybdina*	*Trimmatothelopsisserpentinicola*
*Acarosporamacrospora*	*Myriosporamyochroa*	*Trimmatothelopsisterricola*
*Acarosporanevadensis*	*Myriosporarhagadiza*	
*Acarosporamoenium*	*Myriosporasmaragdula*	

The diversity of described Acarosporaceae in North America does not represent all the family’s diversity. For instance, *Acarosporaprivigna* and *S.pruinosa* are European names of species projected onto North American taxa and concealing unrecorded diversity. The *Acarosporaprivigna* group has at least six undescribed taxa with carbonized epihymenial accretions based on our current research at the Kocourková lab. The *Sarcogynepruinosa* group has at least four undescribed lecideine species on calcareous rock (Knudsen et al. 2023c).

The *Acarosporaprivigna* and *S.pruinosa* groups represent just part of the undescribed diversity of Acarosporaceae in North America. There are several undescribed members of the *A.squamulosa* group (including several taxa mis-identified as *A.peliocypha* or *A.rugulosa*) (Knudsen et al. 2023a). In the *squamulosa* group we only recognize *A.squamulosa* and *A.rugulosa* from the Holarctic flora and *A.ryanii* from the southwestern North American flora. The *Acarosporafuscata* s. lato group are several undescribed brown taxa producing gyrophoric/lecanoric acid. *Acarosporafuscata* s. str. does occur in eastern North America (Knudsen et al. 2022b). But the name has often been misapplied to any brown taxon with gyrophoric/lecanoric acid (Knudsen et al. 2023a, see discussion of *A.agostiniana*). Older reports cannot be accepted without verification. Specimens determined by K. Knudsen as *A.obnubila* should be revised (Knudsen and Kocourková 2023). For instance, the four new species reported from Joshua Tree National Park in this paper were all originally determined as *A.obnubila* (Knudsen et al. 2013).

In the Holarctic lichen flora of North America more species only known from Europe are expected to be discovered like *A.bullata* recently (Brinker and Knudsen 2019). Both *Acarosporacervina* and *A.glaucocarpa* were misapplied to the North American species *S.wheeleri* and *S.canadensis* because they had squamules with white margins and occurred on calcareous rock (Knudsen et al. 2020; Spribille et al. 2023). It is possible, for instance, that *A.cervina* or *A.glaucocarpa* may be found in the Holarctic flora of North America like *S.canadensis* was collected in Romania (Knudsen et al. 2023d).

The American southwest encompasses in the United States the states of Arizona, Nevada, New Mexico, and Utah, most of California, southwestern Texas, and a part of Great Basin Desert extending into Oregon. It contains major deserts: the Chihuahuan, the Mojave, the Sonoran and the Great Basin. The diversity of Acarosporaceae is important for the study of lichen diversity in southwestern U.S.A. and North America. The southwestern U.S.A. is a center of diversity for the family in North America (Magnusson 1930, 1937, 1952, 1956; Knudsen et al. 2021a, 2023a). We reported the diversity of Acarosporaceae in southwestern America as 93 species (Knudsen et al. 2023a). We currently recognize 103 verified species of Acarosporaceae in southwest America which 81.1% of the total species in North America north of Mexico.

Shirley Tucker reported 1,869 described taxa of lichens, allied fungi and lichenicolous fungi from California (Tucker 2014). California needs a new checklist incorporating the Yosemite checklist which reported 584 species of lichens and lichenicous fungi from the national park (Hutten et al. 2013) as well as reports and nomenclatural changes in the literature since 2012. The California Lichen Society has begun this revision [R. Naesborg (SBBG), pers. com.] For California, including the new species described in this paper, we report 62 verified Acarosporaceae (Table 2). There are still several literature and specimen citations of Acarosporaceae reported from California in need of verification (Tucker 2014). The center of diversity of Acarosporaceae in California is in central and southern California (including southern Sierra Nevada Mountains and White Mountains). The Acarosporaceae collected in Yosemite were all species known from southern California except *Acarosporabadiofusca* (Hutten et al. 2013). In southern California *Acarosporaboulderensis* is common and has been identified as *badiofusca* in the past (Knudsen et al. 2014). We verified 62 species of Acarosporaceae in California (Table 2).

**Table 2.** Sixty-two species of described Acarosporaceae are reported from California. Not included in the list are species determined as *Acarosporaprivigna* s. lato (probably three undescribed taxa), *Acarosporafuscata* s. lato (at least two undescribed species), Acarosporacf.squamulosa (several undescribed taxa identified as *A.peliscypha* or sometime *rugulosa*) and Sarcogynecf.pruinosa (at least three undescribed taxa on calcareous rock).

*Acarosporaaffinis*	Along Colorado River	Nash 8464 (ASU)
*Acarosporaalba*	Joshua Tree National Park	Knudsen 13222 (SBBG)
*Acarosporaamericana*	San Luis Obispo Co., Creston	Dart 1352 (SBBG)
*Acarosporabadiofusca*	Yosemite National Park	Knudsen 11703 (YOSE)
*Acarosporaboulderensis*	Mono Co, Inyo NF, Hot Creek	Knudsen 14775 (SBBG)
*Acarosporabrattiae*	Santa Barbara Co, Los Alamos	Bratt 6521 (SBBG)
*Acarosporabrodoana*	San Bernardino Mountains	Knudsen 14703 (SBBG)
*Acarosporadestructans*	Santa Monica Mountains	Hasse (FH)
*Acarosporaelevata*	San Gabriel Mountains	Hasse (FH)
*Acarosporaepilutescens*	Palm Springs	Hasse (W)
*Acarosporaerratica*	White Mountains	Knudsen 16941 (SBBG)
*Acarosporaindistincta*	Joshua Tree National Park	Knudsen 12772 (SBBG)
*A.interpositav.nitidella*	San Bernardino Mountains	Knudsen 16261 (SBBG)
*Acarosporaleavittii*	Granite Mountains	Knudsen 9705 (SBBG)
*Acarosporalendermeri*	San Bernardino Mountains	Lendemer 14917A (NY)
*Acarosporamacrospora*	Joshua Tree National Park	Knudsen 13104 (SBBG)
*Acarosporanevadensis*	Granite Mountains	Knudsen 4386 (SBBG)
*Acarosporanodulosa*	Palm Springs	Hasse (FH)
*Acarosporanovomexicana*	Santa Barabara	Bratt 10314 (SBBG)
*Acarosporaobpallens*	Santa Monica Mountains	Hasse (FH)
*Acarosporaoligospora*	San Jacinto Mountains	Knudsen 1196 (SBBG)
*Acarosporaorcuttii*	San Diego	Orcutt (FH)
*Acarosporanevadensis*	Granite Mountain	Knudsen 4386 (SBBG)
*Acarosporapeltastica*	Joshua Tree National Park	Knudsen 19466 (SBBG)
*Acarosporaradicata*	Joshua Tree National Park	Knudsen 13537 (SBBG)
*Acarosporarobiniae*	Catalina Island	Knudsen 15237 (SBBG)
*Acarosporarosulata*	Joshua Tree National Park	Knudsen 3564 (SBBG)
*Acarosporaschleicheri*	Wildomar, Menifee Hills	Knudsen 3421 (SBBG)
*Acarosporasharnoffii*	Joshua Tree National Park	Kocourková 11119 (SBBG)
*Acarosporasinopica*	Yosemite National Park	Knudsen 11615 (SBBG)
*Acarosporasocialis*	Monterey Co.	Dart 564 (SBBG)
*Acarosporastapfiana*	Joshua Tree National Park	Knudsen 17856 (SBBG)
*Acarosporasuccedens*	Joshua Tree National Park	Knudsen 12876 (SBBG)
*Acarosporasuperfusa*	Joshua Tree National Park	Knudsen 19467 (SBBG)
*Acarosporatejonensis*	Kern Co., Tejon Ranch Conservancy	Knudsen 18288 (SBBG)
*Acarosporathamnina*	Kern Co., Caliente Ranch	Knudsen 15881 (SBBG)
*Acarosporathelococcoides*	Wildomar, Menifee Hills	Knudsen 4356 (SBBG)
*Acarosporaveronensis*	San Bernardino Mountains	Knudsen 17263 (SBBG)
*Glypholeciascabra*	Clark Mountains	Knudsen 11742 (SBBG)
*Myriosporahassei*	Santa Monica Mountains	Knudsen 709 (SBBG)
*Pleopsidiumchlorophanum*	Mount Whitney summit	Hollinger 14965 (hb. Hollinger)
*Pleopsidiumflavum*	San Bernardino Mountains	Knudsen 1185 (SBBG)
*Sarcogyneadscendens*	Santa Ana Mountains	Knudsen 6079 (H)
*Sarcogynearenosa*	San Luis Obispo Co.	Dart 1340 (SBBG)
*Sarcogynebernardinensis*	San Bernardino Mountains	Knudsen 16505 (SBBG)
*Sarcogynecalifornica*	Santa Monica Mountains	Holotype, Hasse (FH)
*Sarcogyneclavus*	Santa Ana Mountains	Kocourková10800 (hb. K&K)
*Sarcogynecoeruleonigricans*	San Jacinto Mountains	Kocourková 11119 (hb. K&K)
*Sarcogynecrustacea*	San Gabriel Mountains	Holotype, Hasse (FH)
*Sarcogynefasciculata*	Joshua Tree National Park	Knudsen 5212 (SBBG)
*Sarcogynehypophaea*	Joshua Tree National Park	Knudsen 13013 (SBBG)
*Sarcogynemitziae*	Joshua Tree National Park	Knudsen 13688 (SBBG)
*Sarcogynenovomexicana*	San Bernardino Mountains	Knudsen 1601 (NY)
*Sarcogyneparadoxa*	Joshua Tree National Park	Knudsen 3620 (SBBG)
*Sarcogyneplicata*	San Gabriel Mountains	Knudsen 1230 (SBBG)
*Sarcogynepusilla*	San Jacinto Mountains	Knudsen 2012 (SBBG)
*Sarcogynesimilis*	Santa Ana Mountains	Knudsen 6035 (SBBG)
*Sarcogyneurceolata*	White Mountains	Tucker 39025 (SBBG)
*Trimmatothelopsiscalifornica*	Monterey Co., Cholame Hills	Dart 577 (SBBG, OBI)
*Trimmatothelopsisoreophila*	San Jacinto Mountains	Knudsen 3459 (SBBG)
*Trimmatothelopsisserpentinicola*	Coon Mountain, Northern California	Carlberg 02937B (SBBG)
*Trimmatothelopsisterricola*	Santa Monica Mountains	Knudsen 5608 (SBBG)

Based on our current results we increase the number of verified Acarosporaceae in New Mexico (*A.stapfiana* and *S.fasciculata*) to 58 species, see Table 1 in Knudsen et al. (2023a). The Joshua Tree National Park has 148 described species of lichens (Knudsen and Kocourková 2023). With the publication of four new species reported from Joshua Tree National Park and the synonymy of *A.obnubila* with *A.elevata*, the total of described lichen species reported is 152 (Knudsen and Kocourková 2023).

These diversity numbers will change as studies of Acarosporaceae in the Southwest of North America continue. As with most fungi, even in the Ascomycota, our knowledge of Acarosporaceae diversity is far from complete (Hawksworth and Lücking 2017).

## Supplementary Material

XML Treatment for Acarospora alba

XML Treatment for Acarospora indistincta

XML Treatment for Acarospora sharnoffii

XML Treatment for Acarospora tejonensis

XML Treatment for Sarcogyne fasciculata

## ﻿Additional information

### Author ORCIDs

Kerry Knudsen https://orcid.org/0000-0001-5419-5729

Jessica Cho-Ah-Ying https://orcid.org/0009-0001-4975-9355

Jana Kocourková https://orcid.org/0000-0001-5511-9752

Eva Hodková https://orcid.org/0000-0002-8337-3253

Jiří Malíček https://orcid.org/0000-0002-3119-8967

Yan Wang https://orcid.org/0000-0002-5950-8904

### Data availability

All of the data that support the findings of this study are available in the main text or Supplementary Information.

## Supplementary materials

Supplementary material 1 Most likehood inference tree Data type pdf Explanation note Most likehood inference tree obtained by phylogenetic analysis using a combined data set of ITS, mtSSU and nLSU sequences of 115 members of Acarosporaceae. *Pycnorasorophora* was used as outgroup. In bold for California are five species new for science and one new report. https://binary.pensoft.net/file/1221233This dataset is made available under the Open Database License (http://opendatacommons.org/licenses/odbl/1.0/). The Open Database License (ODbL) is a license agreement intended to allow users to freely share, modify, and use this Dataset while maintaining this same freedom for others, provided that the original source and author(s) are credited.Kerry Knudsen, Jessica Cho-Ah-Ying, Jana Kocourková, Eva Hodková, Jiří Malíček, Yan Wang

Supplementary material 2 A list of sampled specimens included in molecular phylogeny Data type pdf Explanation note Newly produced sequences are shown in bold. Origin U.S.A. unless stated otherwise. Sequences LSU is included in ITS published Westberg et al. 2015. https://binary.pensoft.net/file/1221234This dataset is made available under the Open Database License (http://opendatacommons.org/licenses/odbl/1.0/). The Open Database License (ODbL) is a license agreement intended to allow users to freely share, modify, and use this Dataset while maintaining this same freedom for others, provided that the original source and author(s) are credited.Kerry Knudsen, Jessica Cho-Ah-Ying, Jana Kocourková, Eva Hodková, Jiří Malíček, Yan Wang
