# Supplementary material for: ﻿The diversity of Acarosporaceae (Acarosporales, Lecanoromycetes) in California
Source: MycoKeys. 2025 Jan 16;112:183–210. doi: 10.3897/mycokeys.112.138580 (PMC11758098; doi:10.3897/mycokeys.112.138580)
Supplement: Supplementary material 1 — Most likehood inference tree [file mycokeys-112-183-s001.pdf]

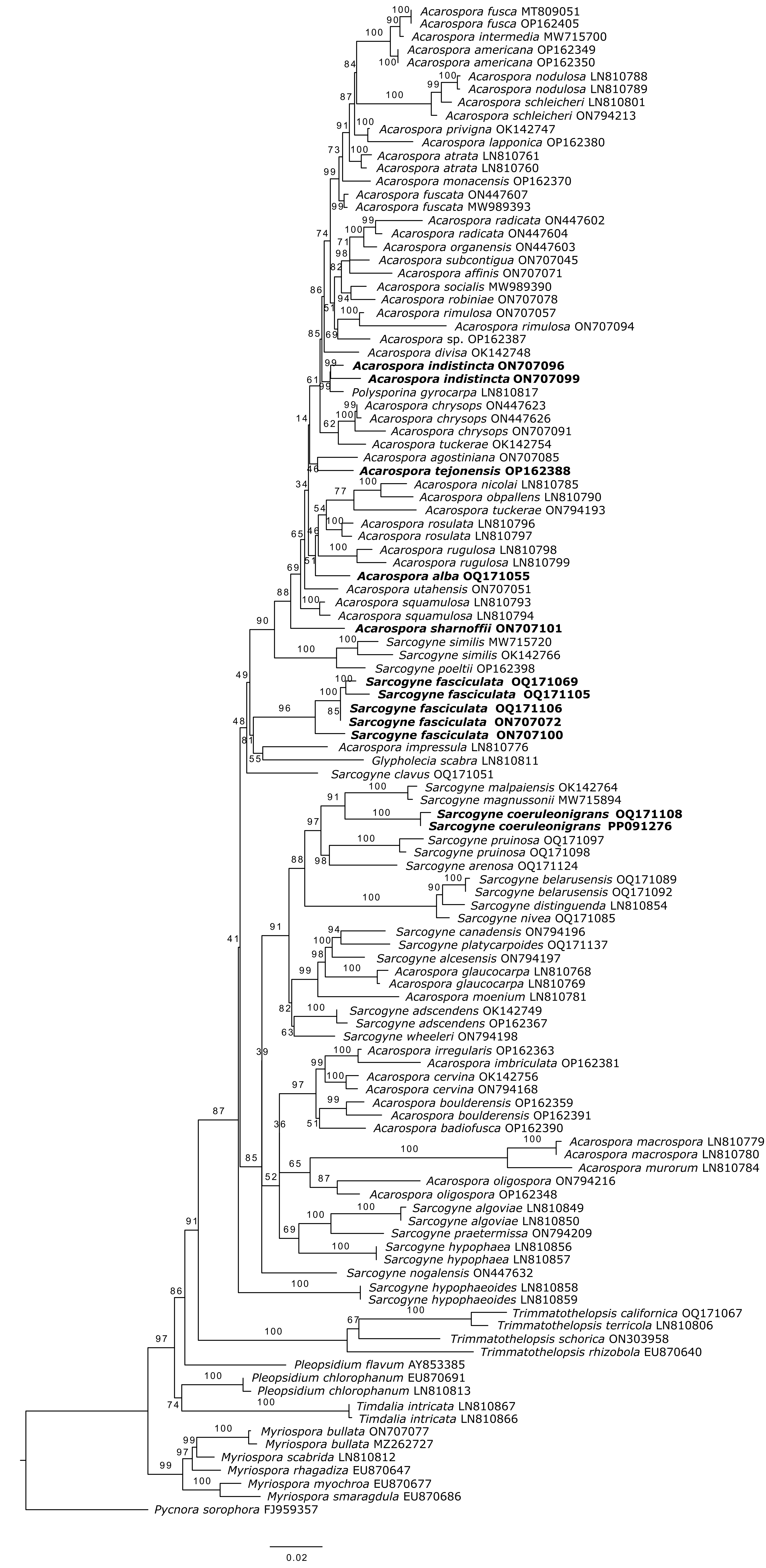

Supplemental Tree1. Maximum likehood tree using a combined data set of ITS, mtSSU and nLSU sequences of 115 members of Acarosporaceae. *Pycnora sorophora* was used as outgroup. In bold for California are five species new for science and one new report.
