# Supplementary material for: ﻿The diversity of Acarosporaceae (Acarosporales, Lecanoromycetes) in California
Source: MycoKeys. 2025 Jan 16;112:183–210. doi: 10.3897/mycokeys.112.138580 (PMC11758098; doi:10.3897/mycokeys.112.138580)
Supplement: Supplementary material 2 — A list of sampled specimens included in molecular phylogeny [file mycokeys-112-183-s002.pdf]

Supplemental Material 2. A list of sampled specimens included in molecular phylogeny. Newly produced sequences are shown in bold. Origin U.S.A. unless stated otherwise. Sequences LSU is included in ITS published Westberg et al. (2015).

**Taxon**	**Origin**	**Sample**	**ITS**	**mtSSU**	**LSU**
Acarospora species	California Joshua Tree	Knudsen 13111.2 (SBBG)	**OP162387**	**OP177771**	**OP216704**
Acarospora admissa	Czech Republic	Malíček 7900 (hb. Malíček)	**ON447564**	**ON367836**	**ON391422**
Acarospora affinis	Mexico	Nash 33959 (hb. K&K)	ON707071	ON715667	
Acarospora agostiniana	New Mexico	Knudsen 19236 (PRM)	OP162389	ON715682	ON725172
Acarospora alba	California Joshua Tree	Knudsen 13181 (SBBG)	**OQ171055**	**OQ184771**	**OQ195825**
Acarospora americana	California	Dart 1526.1 (hb. K&k)	**OP162349**	**OP177737**	**OP216668**
Acarospora americana	California	Dart 750 (hb. K&K)	**OP177738**	**OP177738**	**OP216669**
Acarospora atrata	Norway	Westberg 08-125 (S)	LN810761	LN810886	
Acarospora atrata	Sweden	Arup L02737 (LD)	LN810760	LN810885	
Acarospora badiofusca	Austria	Türk 32318 (GZU)	**OP162390**	**OP177774**	**OP216707**
Acarospora boulderensis	Kansas	Morse 15147 (SBBG)	OP162359	OP177746	OP216677
Acarospora boulderensis	Minnesota	Wetmore 37667 (GZU)	**OP162391**	**OP177775**	**OP216708**
Acarospora cervina	Czech Republic	Kocourková (PRM 952325)	OK142756	OK032141	
Acarospora chrysops	New Mexico	Kocourková 10386 (hb. K&K)	**ON707091**	**ON715686**	**ON725176**
Acarospora chrysops	New Mexico	Knudsen 19385 (hb. K&K)	**ON447623**	**ON367896**	**ON391451**

Acarospora chrysops	New México	Knudsen 19269 (hb. K&K)	**ON447626**	**ON367899**	**ON391454**
Acarospora divisa	New Mexico	Knudsen 19364 (BRY-C)	OK142748	OK032133	
Acarospora fusca	Germany	Schiefelbein 4446 (NY)	MT809052	MT809052	MT809053
Acarospora fusca	Czech Republic	Malíček 14932 (hb. Malíček)	OP162405	OP177790	OP216720
Acarospora fuscata	Norway	Tønsberg 45870 (BG)	**ON447607**	**ON367880**	**ON391440**
Acarospora fuscata	Czech Republic	Kocourková 8499 (S)	MW989393	MW989442	
Acarospora glaucocarpa s. str.	Sweden, Gotland	Westberg SAR08 (LD)	LN810768	LN810893	
Acarospora glaucocarpa s. str.	Sweden, Öland	Westberg, W23 (LD)	LN810769	LN810894	
Acarospora indistincta	California Joshua Tree	Knudsen 5782 (SBBG)	**ON707096**	**ON715691**	**ON725180**
Acarospora indistincta	California Joshua Tree	Knudsen 12772 (SBBG)	**ON707099**	**ON715695**	**ON725184**
Acarospora intermedia	Czech Republic	Knudsen 18342 (hb. K&K)	MW715700	MW715732	MW715723
Acarospora imbriculata	Italy	Buschardt 2187 (B)	OP162381	OP177765	
Acarospora impressula	Norway Oslo	Westberg 08-107 (S)	LN810776	LN810901	
Acarospora irregularis	Czech Republic	Malíček 2128 (hb. Malíček)	OP162363	OP177750	OP216681
Acarospora lapponica	Czech Republic	Bouda (923411 PRM)	**OP162380**	**OP177764**	**OP216698**
Acarospora leavittii	California	Knudsen 9705 (S)	LN810817	LN810942	
Acarospora macrospora	Norway, Oslo	Westberg 08-109 (S)	LN810779	LN810904	

Acarospora macrospora	Sweden, Gotland	Westberg 159 (LD)	LN810780	LN810905	
Acarospora moenium	Sweden	Westberg 09-066 (S)	LN810781	LN810906	
Acarospora monacensis	Czech Republic	Peksa PL BL 2878	OP162370	OP177756	OP216688
Acarospora murorum	Spain	Westberg SCIN014 (S)	LN810784	LN810909	
Acarospora nicolai	Kansas	Morse 16138 (S)	LN810785	LN810910	
Acarospora nodulosa	Spain	Westberg SCIN032 (S)	LN810788	LN810913	
Acarospora nodulosa	Spain, Madrid	Westberg 10-215 (S)	LN810789	LN810914	
Acarospora obpallens	California	Knudsen 9325 (S)	LN810790	LN810915	
Acarospora oligospora	Czech Republic	Kocourková 9006 (hb. K&K)	ON794216	ON787700	
Acarospora oligospora	California	Dart 1424 (hb. K&K)	OP162348	OP177735	
Acarospora privigna	Germany	Kison 4432/3 (hb. K&K)	OK142747	OK032132	ON303622
Acarospora radicata	California Joshua Tree	Knudsen 13537 (SBBG)	**ON447602**	**ON367875**	**ON391435**
Acarospora radicata	California, Joshua tree	Knudsen 13482 (SBBG)	**ON447604**	**ON367877**	**ON391437**
Acarospora rosulata	Norway, Oppland	Westberg 08-193 (S)	LN810797	LN810922	
Acarospora rimulosa	New Mexico	Knudsen 19053 (SBBG)	**ON707057**	**ON715653**	**ON725160**
Acarospora rimulosa	New Mexico, Organ Mountains	Knudsen 19034 (hb. K&K)	**ON707059**	**ON715655**	**ON725162**
Acarospora rimulosa	New Mexico	Kocourková 10336 (hb. K&K)	**ON707094**	**ON715689**	**ON725179**

Acarospora robiniae	California, Santa Ana Mountains	Knudsen 7972 (SBBG)	ON707078	ON715674	ON725169
Acarospora rugulosa	Norway, Telemark	Westberg 08-119 (S)	LN810798	LN810923	
Acarospora rugulosa	Norway	Westberg 10-099 (S)	LN810799	LN810924	
Acarospora schleicheri	California, San Diego	Knudsen 15559 (SBBG)	**ON794213**	**ON787697**	
Acarospora schleicheri	Arizona	Sweat KGS1196 (UPS)	LN810801	LN810926	
Acarospora scottii	Minnesota	Scott 5146 (PRM)	MT809048	MT809049	MT809050
Acarospora sharnoffii	California, Joshua Tree	Sharnoff 4107 (SBBG)	**ON707101**	**ON715697**	**ON725185**
Acarospora socialis	California	Dart 518 (hb. K&K)	MW989390	MW989439	MW989408
Acarospora squamulosa	Sweden, Uppland	Westberg 09-222 (S)	LN810793	LN810918	
Acarospora squamulosa	Norway	Westberg 08-153 (S)	LN810794	LN810919	
Acarospora subcontigua	New Mexico	Knudsen 19366 (SBBG)	**ON707045**	**ON715642**	**ON725148**
Acarospora thamnina	California, Sierra Navada Mountains	Knudsen 15881 (SBBG)	**ON707090**	**ON715685**	**ON725175**
Acarospora tuckerae	Mexico	Huereca 906 (SBBG)	**ON794193**	**ON787675**	
Acarospora utahensis	Colorado	Anderson & Poelt (GZU)	**ON707051**	**ON715648**	**ON725154**
Glypholecia scabra	Norway, Oppland	Westberg 08-232 (S)	LN810811	LN810936	
Myriospora bullata	Germany	Schieftlbein 5321 (B)	ON707077	ON715673	ON725168
Myriospora bullata	Germany	Schiefelbein 4763 (B)	MZ262727	MZ262739	MZ262739
Myriospora myochroa	Sweden, Bohuslän	Westberg 06-051 (LD)	EU870677	EU870729	LN810873

Myriospora rhagazida	Sweden, Bohuslän	Westberg 06-040 (LD)	EU870647	EU870699	LN810875
Myriospora scabrida	Sweden	Santesson 33077a (UPS)	LN810812	LN810937	
Myriospora smaragdula	Sweden	Ågren 345 (UPS)	EU870686	EU870738	LN810878
Pleopsidium chlorophanum	Sweden	Nordin 4439 (UPS)	LN810813	LN810938	
Pleopsidium chlorophanum	Sweden	Nordin 6209 (UPS)	LN810813	LN810938	
Pleopsidium flavum	Austria	Obermayer 5590 (UPS)	AY853385	AY853336	
Pycnora sorophora	Sweden	Hermansson 7903a (UPS)	FJ959357	AY853387	AY853387
Sarcogyne adscendens	California. Santa Ana Mountains	Knudsen 6079 (H)	OP162367	OP177753	OP216685
Sarcogyne adscendens	California	Dart 1230 (SBBG)	OK142749	OK032134	
Sarcogyne alcesensis	Montana	Wheeler 5971 (PRM)	ON794197	ON787680	
Sarcogyne algoviae	Norway, Oppland	Westberg 08-276 (S)	LN810849	LN810975	
Sarcogyne algoviae	Norway, Oppland	Westberg 06-168(S)	LN810850	LN810976	
Sarcogyne arenosa	California	Dart 19949 (SBBG)	OQ171124	OQ184835	OQ195884
Sarcogyne belarusensis	Belarus	Golubkov 80 (H)	OQ171089	OQ184801	OQ195852
Sarcogyne belarusensis	Belarus	Golubkov 73 (PRM)	OQ171092	OQ184804	OQ195855
Sarcogyne canadensis	Montana	Wheeler 5783 (PRM)	**ON794196**	**ON787678**	
Sarcogyne clavus	California Santa Ana Mountains	Kocourková 10880 (hb. K&K)	OQ171051	OQ184767	OQ195822
Sarcogyne coeruleonigrans	New Mexico	Schultz 16242 (HBG)	OQ171108	OQ184820	OQ195870

Sarcogyne coeruleonigrans	California	Kocourková 11119 (hb. K&K)	**PP091276**	**OR754074**	**OR742179**
Sarcogyne distinguenda	Sweden	Westberg 08-305 (S)	LN810854	LN810979	
Sarcogyne fasciculata	California Joshua Tree	Knudsen 5215 (SBBG)	**ON707100**	**ON715696**	
Sarcogyne fasciculata	California	Knudsen 16494.2 (SBBG)	**ON707072**	**ON715668**	**ON725164**
Sarcogyne fasciculata	New Mexico	Kocourková 10848 (PRM)	**OQ171069**	**OQ184781**	**OQ195834**
Sarcogyne fasciculata	New Mexico	Kocourková 10974 (PRM)	**OQ171105**	**OQ184817**	**OQ195867**
Sarcogyne fasciculata	New Mexico	Kocourková 10863 (SBBG)	**OQ171106**	**OQ184818**	**OQ195868**
Sarcogyne fasciculata	New Mexico	Kocourková 10974 (hb. K&K)	**OQ171116**	**OQ184827**	**OQ195876**
Sarcogyne hypophaea	Sweden, Uppland	Westberg SAR198 (S)	LN810856	LN810981	
Sarcogyne hypophaea	Finland	Pykälä 23561 (H)	LN810857	LN810982	
Sarcogyne hypophaeoides	Sweden	Westberg 08-002	LN810858	LN810983	
Sarcogyne hypophaeoides	Norway	Westberg 08-139 (S)	LN810859	LN810984	
Sarcogyne nogalensis	New Mexico	Knudsen 19340 (SBBG)	ON447632	ON367905	ON391460
Sarcogyne malpaiensis	New Mexico	Knudsen 19317.1 (SBBG)	OK142764	OK032149	
Sarcogyne magnussonii	Canada	Freebury 829B (SBBG)	MW715694	MW715694	MW715727
Sarcogyne nivea	Czech Republic	Malíček 14530 (hb. Malíček)	OQ171085	OQ184797	OQ195848
Sarcogyne platycarpoides	Italy	Nimis 34786 (TSB)	OQ171137	OQ184847	OQ195896

Sarcogyne poeltii	Greece	Poelt 78 (GZU)	OP162398	OP177782	OP216714
Sarcogyne praetermissa	Czech Prague	Malíček 9962 (hb. Malíček)	ON794209	ON787693	
Sarcogyne pruinosa	Czech Republic	Malíček 2560 (hb. Malíček)	OQ171097	OQ184809	OQ195860
Sarcogyne pruinosa	Czech Republic	Malíček 6296 (hb. Malíček)	OQ171098	OQ184810	OQ195861
Sarcogyne similis	California	Knudsen 6431 (SBBG)	OK142766	OK032151	
Sarcogyne similis	California	Dart 1332 (SBBG)	MW715720	MW715741	MW715730
Timdalia intricata	Sweden	Westberg P114 (S)	LN810867	LN810992	
Timdalia intricata	Sweden	Westberg SAR92 (LD)	LN810866	LN810991	
Trimmatothelopsis rhizobola	Sweden	Westberg 2994 (LD)	EU870640	EU870692	LN810868
Trimmatothelopsis terricola	California	Knudsen 11216 (S)	LN810806	LN810931	
Trimmatothelopsis schorica	Czech Republic	Kocourková 8980 (hb. K&K)	ON303958	ON303849	ON303963
